# Cross sectional imaging of truncal and quadriceps muscles relates to different functional outcomes in cancer

**DOI:** 10.1016/j.clnu.2018.12.023

**Published:** 2019-12

**Authors:** A.J. MacDonald, J. Miller, M.I. Ramage, C. Greig, N.A. Stephens, C. Jacobi, T. Preston, K.C.H. Fearon, R.J.E. Skipworth

**Affiliations:** a Clinical and Surgical Sciences, University of Edinburgh, Royal Infirmary of Edinburgh, 51 Little France Crescent, Edinburgh EH16 4SA, United Kingdom; b School of Sport, Exercise, and Rehabilitation Sciences, MRC-Arthritis Research UK Centre for Musculoskeletal Ageing Research, NIHR Birmingham BRC, University of Birmingham, B15 2TT, United Kingdom; c Musculoskeletal Diseases Area, Muscle Group, Novartis Pharma AG, Novartis Campus, WSJ- 152.2.72.04, CH-4056, Basel, Switzerland; d Stable Isotope Biochemistry Laboratory, Scottish Universities Environmental Research Centre, The University of Glasgow, East Kilbride, G75 0QF, United Kingdom

**Keywords:** Quadriceps, Truncal muscle, Function, CT, MRI

## Abstract

**Introduction:**

Following the consensus definition of cancer cachexia, more studies are using CT scan analysis of truncal muscles as a marker of muscle wasting. However, how CT-derived body composition relates to function, strength and power in patients with cancer is largely unknown.

**Aims:**

We aimed to describe the relationship between CT truncal (L3) skeletal muscle index (SMI) and MRI quadriceps cross sectional area with lower limb strength, power and measures of complex function.

**Methods:**

Patients undergoing assessment for potentially curative surgery for oesophagogastric or pancreatic cancer were recruited from the regional upper gastrointestinal (UGI) or hepatopancreaticobiliary (HPB) multi-disciplinary team meetings. Maximum Isometric Knee Extensor Strength (IKES) and Maximum Leg Extensor Power (Nottingham Power Rig) (LEP) were used as measures of lower limb performance. Both Sit to Stand (STS) and Timed Up and Go (TUG) were used as measures of global complex muscle function. Muscle SMI was measured from routine CT scans at the level of the third lumbar vertebrae (L3) and MRI scan was used for the assessment of quadriceps muscles. Linear regression analysis was performed for CT SMI or MRI quadriceps as a predictor of each measure of performance.

**Results:**

Forty-four patients underwent assessment. Height and weight were significantly related to function in terms of quadriceps power, while only weight was associated with strength (P < 0.001). CT SMI was not related to measures of quadriceps strength or power but had significant association with more complex functional measures (P = 0.006, R^2^ = 0.234 and 0.0019, R^2^ = 0.175 for STS and TUG respectively). In comparison, both gross and fat-subtracted measures of quadriceps muscle mass from MRI were significantly correlated with quadriceps strength and power (P < 0.001), but did not show any significant association with complex functional measures.

**Conclusion:**

CT SMI and MRI quadriceps have been shown to reflect different aspects of functional ability with CT SMI being a marker of global muscle function and MRI quadriceps being specific to quadriceps power and strength. This should therefore be considered when choosing outcome measures for trials or definitions of muscle mass and function.

## 1 Introduction

Muscle mass may be measured during muscle wasting as a marker of disease severity, progression or response to treatment. As currently there are no quantitative surrogate biochemical markers for muscle wasting, evaluation relies on direct assessment of muscle mass. Typically, these assessments measure limb muscle mass by DXA or MRI, or truncal muscle mass by CT [which can be indexed for height - skeletal muscle index (SMI)]. These muscle groups may have different biological responses to cancer cachexia, and thus may make different relative contributions to reduced physical function, perhaps the most important clinical sequela of muscle wasting in cancer cachexia or aging.

Studies of muscle size and function typically assess strength or power along with CT SMI and volume in specific muscle groups. This relationship is well studied in healthy individuals [1], [2] and in aging [3], with a clear correlation seen between muscle mass and strength [4], [5]. Despite such correlations, there is significant variation in muscle strength and power between individuals, not explained by simple differences in sex or lean mass [5]. This variation has been extensively studied in aging where age-related changes in muscle quality may contribute to changes in muscle function in terms of strength and power [6]. Although SMI is commonly reported in the assessment of cancer cachexia, the relationship of muscle area or volume with strength or power is not well described in this patient group. This question is of particular importance as pain, fatigue and symptoms associated with cancer or cancer treatment, as well as alterations in intramuscular fat, will all affect the functional performance of these patients [7]. Therefore, in studies of cancer patients, it is not always practical or appropriate to undertake extensive investigation that adds to the burden of routine cancer investigation or treatment, particularly with regard to measures requiring significant physical exertion. Use of routine diagnostic CT scans is cheaper, quicker, reduces patient burden and also allows for retrospective analysis [8]. This modality is now extensively used but has not been validated against function, limb strength and power in patients with cancer.

The commonly studied muscle groups include the muscles of the lower limb, thigh and quadriceps, or the upper limb biceps area or forearm muscles involved in hand grip strength [9], [10]. These muscles or muscle groups are chosen as they are known to be functionally important in common actions such as chair rising and stair climbing, or may contribute to clinically significant events e.g. falls in the elderly [11]. Additionally, complex functional measures such as TUG and STS times are related to both muscle function and to disability and risk of falls [12]. These measures of function may be considered in a spectrum of complexity whereby “simple” measures aim to assess a single muscle or muscle group such as isometric knee extensor strength (IKES). Dynamic measures such as Leg extensor power (LEP), where the limb moves during the measure are more prone to recruit additional muscle groups and may provide a less “pure” measure of an individual muscle group. In comparison, the Sit to Stand (STS) test is a more complex functional measure of muscle strength and power that also involves coordination of lower limb and truncal muscles, whereas the Timed Up and Go (TUG) is more “complex” still involving both muscle strength, power, coordination and balance.

Therefore, we aimed to describe the relationship between CT truncal (L3) skeletal muscle index (SMI) and MRI quadriceps cross sectional area with lower limb strength, power and measures of complex function.

## 2 Methods

### 2.1 Patient recruitment

Patients were recruited from the regional upper gastrointestinal (UGI) or hepatopancreaticobiliary (HPB) multi-disciplinary meetings prior to undergoing potentially curative surgery for oesophagogastric or pancreatic cancer. Ethical approval was granted by the Lothian research ethics committee. Written informed consent was obtained. Cachexia was defined according to the consensus definition [13]. All patients who took part in the study were deemed fit enough to undergo major resectional surgery. Patients were excluded if they had significant musculoskeletal problems limiting their ability to perform the tests. Isometric tests of truncal muscle strength were not performed. Biodex systems do exist to measure isometric truncal muscle function but have not been validated in this patient group.

### 2.2 Maximum Isometric Knee Extensor Strength (strain gauge) (IKES)

IKES was measured with a strain gauge and recorded with a strain meter. Three separate measurements (Newtons) were obtained for each limb and the highest value from the dominant limb used in subsequent analysis. The coefficient of variation for IKES is 6.9% for a single session and 10% across sessions occurring over several days [14].

### 2.3 Maximum Leg Extensor Power (Nottingham Power Rig) (LEP)

LEP was measured using the Nottingham Power Rig. The instrument consists of an adjustable seat and large foot pedal connected to a flywheel. The final velocity of the flywheel was used to calculate the average power output (Watts) during a single maximal thrust of the lower limb. The process was repeated five times with each limb and the highest value from the dominant limb used in subsequent analysis. The coefficient of variation for repeated tests of leg extensor power measured using the Nottingham power rig in healthy individuals is 8.7% [14].

### 2.4 Measures of complex function

TUG was measured in conjunction with the STS test by two observers as per previous publications [15], [16], [17].

### 2.5 Imaging analysis

CT imaging was chosen for this study as it is now considered the gold standard for body composition analysis. It is easily accessible in this patient group as it is routinely performed as part of the assessment process for cancer patients. CT scans however do not encompass imaging of the whole body and therefore MRI quadriceps was undertaken. Although not widely used in cachexia clinical trials before MRI quadriceps has been used successfully in the Formoterol trial [18].

SMI was measured from routine CT scans performed within 42 days of functional measurement and prior to any surgical intervention or neoadjuvant chemotherapy. Digitally stored CT images completed with a spiral CT were analysed as described previously [19], [20]. Cross-sectional area for muscle was normalized for stature (cm^2^/m^2^) and a lumbar SMI computed. SMI cut-offs for low SMI were based on a CT-based study of cancer patients by Martin et al. [10]. Cachexia was classified according to the consensus definition by Fearon et al. (>5% weight loss or >2% weight loss and low muscularity) [13]. Skeletal muscle density (SMD) was assessed by the mean radiological muscle attenuation of all muscle visible at the L3 level, measured in Hounsfield Units (HU). The HU scale is a radiological scale describing the density of tissues on CT scans; lower mean muscle attenuation indicates less dense muscle tissue with more lipid infiltration [21]. There is currently no consensus for the upper and lower HU cut-offs used to characterize muscle attenuation. In previous studies we have used mean HU for skeletal muscle values below 39.5 (two standard deviations below a normal healthy cohort) to classify low muscle attenuation [22].

MRI was performed on the day of functional assessment. The cross-sectional area (CSA) of muscle in each image was quantified off-line using ANALYZE 8.0 (Mayo Clinic, Rochester, USA) by a manual delineation technique. An automated in house programme based on k-means clustering was applied to the manually segmented image [23]. The purpose of this was to exclude areas of fat lying within the muscle or between muscle groups to provide an objective measure of healthy muscle tissue.

### 2.6 Statistical analysis

Linear regression analysis was performed for CT SMI or MRI quadriceps CSA as a predictor for each measure of function. Strength, power, STS and TUG were correlated with variables known to be associated with function: SMI, SMD, CRP, weight, height and weight loss. Statistical significance was set at a two-tailed p value of ≤0.05. Associations are described with r^2^ values and uncorrected p values for each comparison. P values required for significance after Bonferroni correction for multiple comparisons are displayed for each analysis. Kolmogorov–Smirnov test was used to assess for normality of distribution of data. As this was an exploratory study a sample size calculation was not performed.

## 3 Results

Forty-four (16 female) patients underwent functional assessment. Due to patient frailty and availability IKES was unavailable in three patients, MRI quads in 14, CT in 10, STS and TUG in 4. Characteristics of the overall group are described in Table 1. Nineteen patients had low muscularity on CT scan and 28 were cachectic according to the consensus definition.

**Table 1:** Group characteristics for patients with upper GI cancer undergoing cross sectional imaging and functional assessment.

	Descriptive Statistics		
	Range	Mean	Std. Deviation
Age (years)	39–88	63.34	11.03
Height (M^2^)	1.52–1.90	1.70	0.09
Weight (kg)	48.80–121.70	74.26	15.94
Self-reported weight change (%)	−43.8–10.9	−6.7	9.3
CRP (mg/L)	1–172	20	40
IKES (N)	91.5–548.0	262.9	95.0
LEP (W)	39.0–270.0	101.5	49.0
STS (s)	0.30–0.98	0.63	0.17
TUG (s)	4.21–12.53	7.02	1.64
MRI Quads CSA (cm^2^/m^2^)	12.59–25.62	18.35	3.37
MRI Quads k-means CSA (cm^2^/m^2^)	9.00–23.97	15.38	3.44
CT SMI (cm^2^/m^2^)	34.38–58.40	44.51	6.10
Muscle mean HU	20–55	39.42	8.39

On simple linear regression (p = 0.001 was considered significant to account for multiple comparisons after Bonferroni correction), height and weight were significantly related to function in terms of quadriceps power, whilst only weight was associated with quadriceps strength. These associations could be expected and provide the rationale behind correction of strength and power for stature where they are used independently of other body composition measures [24]. Age, CRP and weight-loss showed weak associations with complex measures of function, but with the exception of TUG with age, these associations lost significance after correction for multiple comparisons (Table 2).

**Table 2:** Regression analysis of patient characteristics with functional measures.

	Height (m)		Weight (Kg)		Age (years)		CRP (mg/l)		Weight change	
	R^2^	P	R^2^	P	R^2^	P	R^2^	P	R^2^	P
IKES (n = 41)	0.049	0.166	0.287	**<0.001**	0.001	0.861	0.113	0.032	0.116	0.029
LEP (n = 44)	0.225	**0.001**	0.199	0.002	0.091	0.049	0.051	0.140	0.061	0.106
STS (n = 40)	0.003	0.748	0.000	0.983	0.101	0.045	0.050	0.163	0.087	0.065
TUG (n = 40)	0.003	0.740	0.000	0.941	0.268	**0.001**	0.162	0.010	0.000	0.891

Significant at p = 0.001 (IKES = Isometric Knee Extensor Strength, LEP = Leg Extensor Power, STS = Sit To Stand time, TUG = Timed Up and Go).

Bold represents statistically significant.

CT SMI was not related to measures of quadriceps strength and power (IKES, LEP) (Table 3), but was significantly associated with more complex functional measures (Table 3) (significance at p ≤ 0.025 for multiple comparisons). No relationship was seen between any measure of muscle performance and CT-derived SMD (Table 3).

**Table 3:** Association of SMI of L3 muscle with quadriceps (Isometric Knee Extensor Strength and Leg Extensor Power) and complex (Sit to stand, Timed Up and Go) function.

Quadriceps muscle strength and power	Truncal (L3) SMI		Truncal (L3) Muscle HU	
	R^2^	P	R^2^	P
Dominant IKES	0.068	0.150	0.032	0.325
Dominant LEP	0.081	0.103	0.014	0.509
**Complex function (N = 31)**				
STS	0.234	**0.006**	0.006	0.672
TUG	0.175	**0.019**	0.103	0.078

Significant at p = 0.025.

Bold represents statistically significant.

Both MRI gross and fat-subtracted measures of quadriceps muscle mass were significantly correlated with quadriceps strength and power (Table 4). No association was seen with the more complex functional measures (Table 4) (significance at p = 0.025)

**Table 4:** Association of height adjusted MRI measures of quadriceps muscle cross sectional area with quadriceps function (Isometric Knee Extensor Strength, Leg Extensor Power).

Quadriceps muscle strength and power N = 28	Quads Muscle CSA (cm/m2) N = 30		Quads K-means muscle (cm/m2) N = 25	
	R^2^	P	R^2^	P
Dominant IKES	0.399	**<0.001**	0.412	**0.001**
Dominant LEP	0.242	**0.006**	0.346	**0.002**
Complex function				
STS	0.024	0.436	0.069	0.227
TUG	0.026	0.409	0.160	0.058

Significant at p = 0.025.

Bold represents statistically significant.

**Fig. 1:**
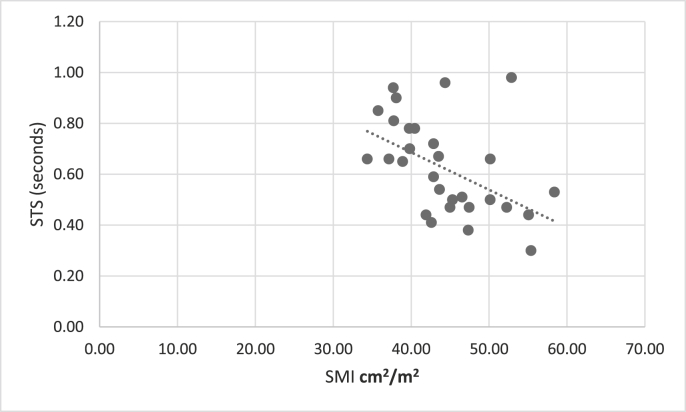
Scatter chart to show correlation between STS and SMI.

**Fig. 2:**
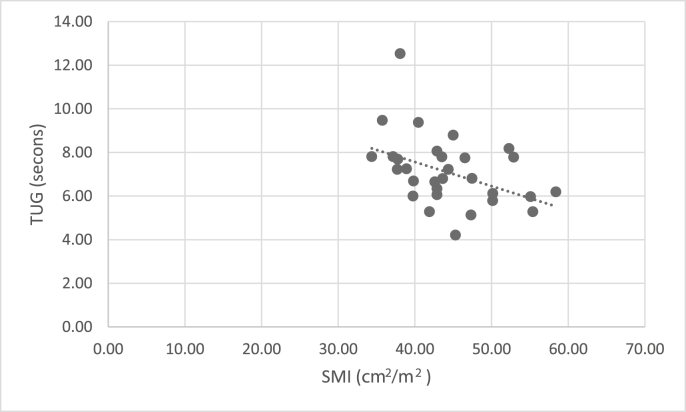
Scatter chart to show correlation between CT SMI and TUG.

**Fig. 3:**
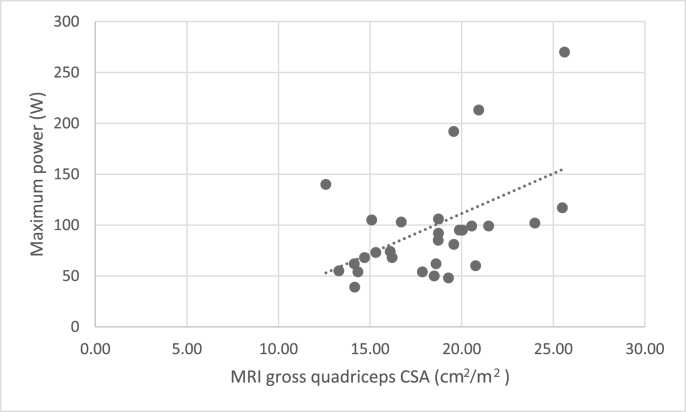
Scatter chart to show correlation between MRI gross quadriceps CSA and power.

**Fig. 4:**
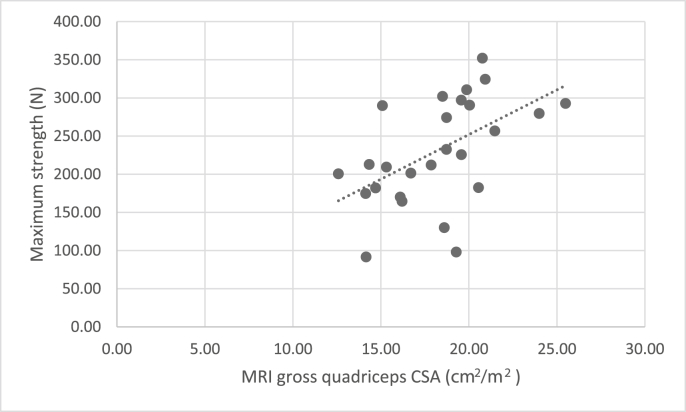
Scatter chart to show correlation between MRI gross quadriceps CSA and maximum strength.

Significant correlations are shown in Fig. 1, Fig. 2, Fig. 3, Fig. 4.

## 4 Discussion

Measures of muscle mass from CT SMI and MRI quadriceps both appear to relate to performance though measures from the two different anatomical locations reflect different functional abilities in this group of patients with cancer. This may be of great importance in the design of future clinical trials. Previous trials have failed as they have been able to demonstrate increases in lean mass but not in functional outcomes [25]. This failure may have been because specific functional read outs such as hand grip strength were used rather than a more in depth assessment of muscle function using various short physical performance tests.

As might be expected, MRI-estimated quadriceps size corrected for stature correlates with quadriceps strength and power. This observation appears to be true whether the gross or fat-subtracted area is used. This finding is consistent with previous studies in healthy individuals where quadriceps CSA correlates with knee extensor strength in healthy young men (r = 0.59) and women (r = 0.51) [5]. The data presented here suggest that fat subtracted CSA could possibly be a more relevant measure as it appears that similar or greater variance (r^2^) is described by the fat subtraction method despite smaller numbers in this group (25 vs 30 in the gross CSA group) and would be consistent with the principle that fat infiltration of muscle is an important influence on muscle function [26]. However, this finding was not reproduced when using HU/SMD as a measure of muscle fat infiltration on CT. CT-derived SMD differs from MRI fat-subtracted muscle area in that although macroscopic adipose tissue is excluded during analysis of CT scans, microscopic or intramyocellular deposits still remain [27]. SMD has previously been shown to correlate with muscle function in terms of mobility limitation in healthy older people [28], but this finding was not reproduced in the present study in terms of strength, power or complex tests of global muscle function. Other factors such as peripheral oedema and gross alteration in hydration status may be present in patients with cancer that can also influence skeletal muscle HU and diminish the strength of this association [29]. With PET-CT now becoming more widely available and used as part of routine cancer staging future studies will be able to compare different muscle groups using the same imaging technique.

MRI quadriceps did not correlate with outcome in complex tests (i.e. STS and TUG). At first glance, this finding may seem surprising as quadriceps strength is normally the strongest predictor of STS times in healthy individuals [28]. However, in community dwelling older people, knee extensor strength explains only 16.5% of the variance in the STS test [30] with other factors such as visual contrast sensitivity, lower limb proprioception, peripheral tactile sensitivity, reaction time, and scores on the Short-Form 12 Health Status Questionnaire pain, anxiety, and vitality scales also contributing. In previous studies, serum CRP concentration has been unfavourably related to physical performance, even within low ranges [31]. In the present study, CRP also correlated with some measures of performance (namely, IKES and TUG) suggesting that systemic inflammation may play a role in patient functional limitation, but these significant associations were lost when correcting for multiple comparisons.

The measures of CT SMI differed from the measures of MRI quadriceps in their associations with performance. As thigh muscle cross-sectional area and L3 muscle cross-sectional area both correlate strongly with whole body muscularity [32], it might be assumed that they should correlate directly with each other and therefore equally with quadriceps strength and power. This assumption was not found to be the case in our dataset. CT-SMI was a predictor of STS and TUG. Both the STS and TUG tasks require the use of a combination of lower limb and trunk motion initiated by erector spinae and involving rectus abdominis in addition to quadriceps and gluteal muscles [32]. Trunk muscles also make a significant contribution to balance and coordination. When weight is shifted, the trunk responds to counteract the change in the centre of gravity, maintaining postural control. Effective trunk muscle function is essential for balance, transfers, gait, and the range of activities in daily living [33]. Trunk muscle fatigue impairs balance and functional task performance [34], [35] and makes an equal or greater contribution than limb muscle strength [32], [33], [34], [35]. As a result, in disease states with muscle wasting, CT SMI may be a better surrogate measure of global functional impairment and disability than MRI quadriceps.

There were some important limitations to this study. The K-means processing was observed to fail on several scans, either due to low contrast across the scan as a whole or due to contrast gradients on individual slices indicating that using MRI as a marker of muscularity instead of CT can be more difficult. IKES was unavailable in three patients. Patients with cancer cachexia can be very frail and often struggle performing tasks asked of them. This is a very important point to consider when deciding on the best functional assessment for studies in order to ensure all are able to participate. The ability to use imaging techniques as measures of patient function therefore are perhaps more appropriate in some settings. We have previously shown that sexual dimorphism exists in skeletal muscle mass, fibre type and size and in response to neoplasm in this patient group [24]. Sex specific analysis was not performed due to the small numbers in this study but this is an important point to consider in the design of future trials. There were outliers observed (e.g. few people with high muscularity on CT had some of the longest STS times) this maybe in part due to an unrecognised co-morbidity preventing them from completing the test as required.

## Conclusion

Both CT SMI and MRI quadriceps are potentially valid markers of muscle function. However, these measures appear not to be interchangeable as they reflect different aspects of functional ability with CT SMI being a better marker of global muscle function. The mass of trunk muscles may play a different role in complex movements compared with the mass of lower limb muscles. Consideration of these differences is essential when selecting measures of muscle mass or function for use in definitions of muscularity in wasting conditions, or for use as outcome measures in studies of wasting conditions. Further studies are required to ascertain the best outcome measure.

## Ethical statement

The study was approved by the Lothian Research Ethics Committee and was therefore been performed in accordance with the ethical standards laid down in the 1964 Declaration of Helsinki and its later amendments. All patients gave written consent prior to inclusion.

## Funding

This study was supported by Cancer Research UK (grant no. C1128/A7309).

## Author's statement

AM, CG and NS undertook the patient assessments. JM, MR and AM wrote the manuscript. TP, CJ and RS critically revised the manuscript. KF designed the project. RS and AM gave final approval for the version to be published.

## Conflicts of interest

The authors declare no conflicts of interest.
